# Application of acupuncture and moxibustion for tic disorders: An overview of systematic reviews and meta-analysis

**DOI:** 10.1097/MD.0000000000046506

**Published:** 2025-12-12

**Authors:** Xiaoqian Li, Lei Xu, Huiling Liang, Jinlan Peng, Guozheng Qin, Lisheng Wan

**Affiliations:** a Shenzhen Children’s Hospital, Guangzhou University of Chinese Medicine, Guangzhou, China; b Yunnan University of Chinese Medicine, Kunming, China; c The First Affiliated Hospital of Yunnan University of Chinese Medicine, Kunming, China.

**Keywords:** acupuncture and moxibustion, effectiveness, meta-analysis, randomized controlled trial, systematic review, tic disorder

## Abstract

**Background:**

Tic disorder (TD) is a neuropsychiatric disorder that often begins in childhood or adolescence. Acupuncture and moxibustion are alternative therapies that have been demonstrated to be effective in the treatment of TD. Over the past few decades, several systematic reviews (SRs) and meta-analyses have reported the effectiveness of acupuncture and moxibustion in treating patients with TD. However, the quality of these SRs varies.

**Methods:**

Ten electronic databases were searched. Two researchers performed independently in study selection, data extraction, and evaluation. Methodological quality was assessed by A Measurement Tool to Assess Systematic Reviews-2. Evidence levels were assessed using the Grading of Recommendations Assessment, Development, and Evaluation approaches.

**Results:**

In total, 12 studies were included, comprising 106 randomized controlled trials and 7973 patients. In A Measurement Tool to Assess Systematic Reviews-2, most of the SRs were of low or critically low levels since they had more than 1 critical deficiency. In the Grading of Recommendations Assessment, Development, and Evaluation system, 10 outcomes were valued as very low level, 30 as low, 24 as moderate level, and 0 as high level. Most SRs meet the potential benefits of acupuncture and moxibustion for TD. Interestingly, no serious events were shown in these studies.

**Conclusion:**

This overview, through analysis of 12 studies, shows that acupuncture and moxibustion were more effective than the conventional Western medicine treatment for TD. Nevertheless, given the subpar methodological quality of the reviews, we cannot draw a more definitive conclusion. Hence, we need more research to improve methodological and reporting quality to yield more robust evidence.

## 1. Introduction

Tic disorder (TD) is a neurodevelopmental disorder that often occurs in childhood or adolescence and is characterized by rapid, sudden, repetitive, involuntary movements (motor tics) and vocalizations (phonic tics).^[1]^ It is generally known that the vast majority of children (79%) have one or more psychological and behavioral disorders, with attention deficit hyperactivity disorder being the most common, accounting for nearly 60%, followed by compulsive disorder.^[2]^ Based on the clinical characteristics and the duration of the disease course, these conditions can be divided into 5 types, namely Tourette Syndrome (TS), chronic TD, provisional TD, undifferentiated TD, and other specific TDs, with the first 3 being more common.^[3]^ The onset age of TD is between 2 and 21 years old, with the most common age group being 5 to 10 years old, however, the most severe cases occur between 10 and 12 years old. It is significantly more prevalent in males than females, with a male-to-female ratio of (2–3.5) to 1.^[4]^ Yet, there are currently no specific diagnostic criteria for TD. In the clinic, its diagnosis and classification are only based on the clinical symptoms. In addition, laboratory tests and imaging examinations show nonspecific changes, so they can only be used for diagnostic assistance. The most commonly used assessment scale currently is the Yale Global Tic Severity Scale (YGTSS).^[5]^

Currently, the specific etiologies and pathogenesis of TD remains inconclusive. Accumulating evidence has suggested that it is association with abnormalities in the cortical-striatal-thalamic-cortical circuit, which is closely linked to neurotransmitter abnormalities, infections and immunity, genetic factors, trace elements, and pregnancy-related factors. Hence, Western medicine mainly treats this disease through education and behavioral interventions, medication, and brain stimulation therapy in clinic. Both European clinical guidelines^[6]^ and American clinical guidelines^[7]^ considered Behavior Therapy (BT) as the first-line intervention for TD. However, BT is not effective or feasible for all patients for a significant portion of patients require medication treatment alone or in combination with behavior therapy, indicating that it’s still a lack of effective clinical therapeutic drugs.^[8–13]^

Over the past few decades, the application of complementary and alternative medicine (CAM) as adjunct therapy of TD shown a sharp increase and acquired good effects. It’s reported that, in the United States, 20% to 40% of children use CAM, with the CAM usage rate among adults reaching as high as 62%. Among them, the evidence showed 41% of respondents have used 2 or more CAM therapies in the past 12 months.^[14]^

AT, a principal modality within CAM, is widely applied in the treatment of TD. Based on traditional Chinese medicine theory, the core mechanism of AT for TD involves regulating meridian qi and blood.^[15]^ Modern research indicates that AT primarily ameliorates TD by modulating the dopamine system.^[16]^ Needling at acupoints Baihui (GV20) and Yintang (GV29) reduces dopamine concentrations in the striatum, substantia nigra pars compacta, and prefrontal cortex, suppresses D1R and D2R receptor expression, and decreases tyrosine hydroxylase activity in the substantia nigra pars compacta, thereby alleviating stereotypic behaviors.^[16]^ Clinical studies confirm that AT significantly reduces YGTSS scores, effectively improves both motor and vocal tics.^[17,18]^ Commonly used acupoints include Baihui (GV20), Fengchi (GB20), and Taichong (LR3).^[19]^

Although numerous systematic reviews (SRs) and meta-analyses on AT for TD exist, significant heterogeneity is evident in their methodological quality and the strength of the evidence. Therefore, this study aims to systematically evaluate the methodological quality and evidence strength of existing SRs/meta-analyses to provide evidence-based support for the clinical application of AT in TD management.

## 2. Materials and methods

### 2.1. Study registration

The protocol has been registered on the OSF (Open Science Framework, https://osf.io/) platform with registration number DOI:10.17605/OSF.IO/EUCAK.

### 2.2. Literature retrieval

We searched 10 databases, including the English, Chinese, and Korean databases of PubMed, EMBASE, the Cochrane Library, Web of Science, MEDLINE, KCI-Korean Journal Database, Chinese National Knowledge Infrastructure, Wanfang Databases, China Science and Technology Journal Database (VIP), and the Chinese biomedical literature service system (CBM), from inception to November 2023. There were no restrictions on the language or the publication status.

The search terms we used are as follows: (“tic disorders” OR “tic” OR “Tourette’s disease” OR “Tourette Syndrome”) AND (“electro-acupuncture” OR “electroacupuncture” OR “auricular acupuncture” OR “moxibustion” OR “acupressure” OR “acupuncture points” OR “acupuncture”) AND (“systematic review” OR “meta-analysis” OR “meta analysis”). Additional studies were identified through the reference lists in the included SRs.

### 2.3. Eligibility criteria

#### 2.3.1. Types of studies

The systematic review and meta-analysis of randomized controlled trials assessing acupuncture for treating TDs meet the inclusion criteria, with no language restrictions imposed.

#### 2.3.2. Types of participants

Individuals diagnosed with TDs, aged under 18, regardless of gender, without limitations on disease staging or symptoms.

#### 2.3.3. Types of interventions and comparators

It includes acupuncture techniques such as filiform needles, electroacupuncture, scalp acupuncture, auricular therapy, and other adjunct therapies, as well as moxibustion studies for treating TDs. Studies comparing the combination of acupuncture with another effective treatment method to the use of other effective treatment methods alone are also eligible. The eligible control groups include conventional standard medications and psychological and behavioral therapies.

#### 2.3.4. Types of outcome measures

The main outcomes measured are the clinical effective rate (including total effective rate, cure rate, and clinical symptom scores) and YGTSS. The incidence of adverse reactions is considered a secondary outcome.

### 2.4. Exclusion criteria

SRs/MAs with non-randomized controlled trial designs; SRs/MAs involving laser acupuncture, acupoint injection, or transcutaneous nerve electrical stimulation; SRs/MAs comparing the efficacy of different acupuncture methods; and SRs/MAs that did not synthesize original data.

### 2.5. Studies selection and data extraction

The 2 authors independently conducted the screening process. The search results were imported into EndNote 20 software to remove duplicate studies. Integrating the inclusion and exclusion criteria, and reviewing the titles and abstracts of the studies, a preliminary assessment of potential evidence was conducted. Subsequently, a meticulous review of these studies was performed for final selection. After determining the final inclusion of systematic reviews and meta-analyses (SRs/MAs), data extraction was carried out independently by the 2 authors (LX and XQL). Basic information such as authors, publication dates, literature search dates, number of databases searched, number of included RCTs, and total sample size was extracted from the studies. Additionally, data on intervention measures, quality assessment tools, overall risk of bias, effect estimates of primary outcomes (meta-analysis), and study conclusions were also extracted. Any conflicts were resolved through discussion with a third author (LSW) during the process.

### 2.6. Quality assessment

Two authors (LXQ and XL) worked independently during the screening process. Two authors (LXQ and XL) used the Assessing the Methodological Quality of Systematic Reviews-2 (AMSTAR-2) tool^[20]^ to assess the methodological quality of each included SRs/MAs. Differences in research assessment were resolved through discussion and consultation with the third author (LSW).

AMSTAR-2 has 3 appraisal categories: “yes,” “partial yes,” or “no,” with 16 approved items, serving as a tool for objective assessment of SRs/MAs. The overall confidence in the results of SRs/MAs is rated as the followed 4 categories: “high” (no flaws or 1 noncritical flaw); “moderate” (more than 1 noncritical flaw); “low” (one critical flaw, with or without noncritical flaws); and “very low” (more than 1 critical flaw, regardless of noncritical flaws). Considering that AMSTAR-2 does not generate an overall “score” and may obscure some key weaknesses of SRs/MAs, a thoughtful judgment process was used to interpret the results of AMSTAR-2 until a consensus was reached on the overall methodological quality of the included SRs/MAs.

### 2.7. Quality of evidence evaluation

The quality of evidence for each outcome was determined using the grading ofrecommendations assessment, development, and evaluation (GRADE) grading methods.^[21]^ The GRADE approach categorizes the quality of evidence into 4 levels: high, moderate, low, and very low. Based on randomized controlled trials, evidence is initially given a high rating, then maybe downgraded, or even upgraded, due to the following reasons: risk of bias; indirectness of evidence; heterogeneity or inconsistency of results; imprecision of results; and publication bias.

Two reviewers (LXQ and XL) independently analyzed the evidence and its associated outcomes. Any factors that could potentially impact the upgrading or downgrading of the quality of evidence will be thoroughly explained to ensure transparent and reliable results. Any discrepancies will be discussed and resolved with the assistance of the third author (LSW).

### 2.8. Data analysis

Due to the overlap of data with RCTs, we did not conduct a quantitative analysis of SRs/MAs. However, we conducted a qualitative analysis and provided a summary. We also calculated the effectiveness of the interventions. The methodological quality of SRs/MAs and the quality of evidence for the results are also included in the table. We analyzed the frequency of acupuncture points used in the original studies included in the SRs/MAs, the interventions employed, and adverse events.

## 3. Results

### 3.1. Study inclusion

**Figure 1.**
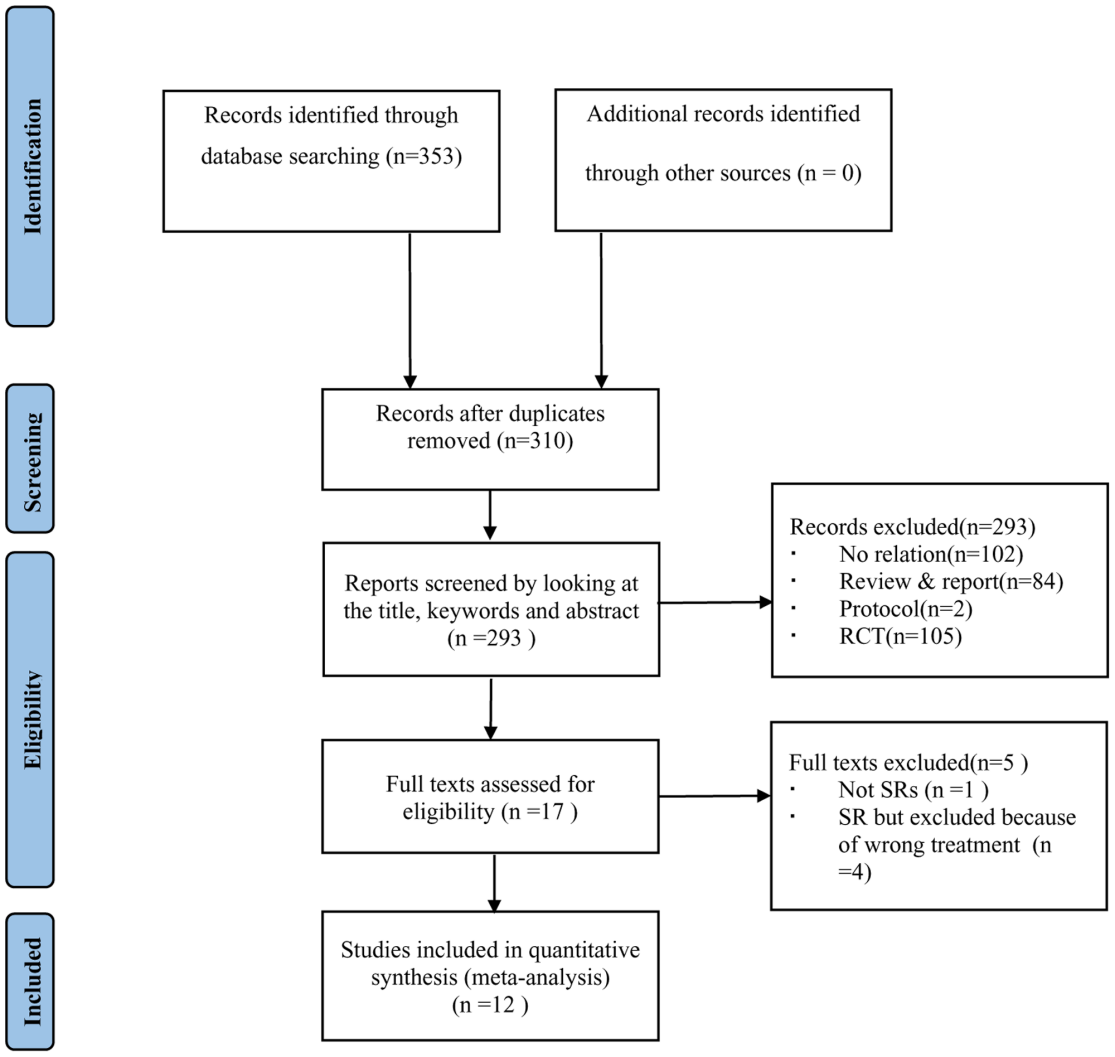
Flowchart of study selection.

We retrieved a total of 353 studies and removed 43 duplicate studies. Then, through further screening titles and abstracts, 293 studies unrelated to the topic were excluded. Finally, after full-text assessments, we selected 12 studies for analysis (Fig. 1).

### 3.2. Characteristics of included studies

All SRs/MAs were released between 2015 and 2023, with 11^[17,22–31]^ coming from China and 1^[32]^ from South Korea (Table 1). Among them, there are a total of 8 articles in Chinese^[22–29]^ and 4 articles in English.^[17,30–32]^ All the included SRs/MAs contain RCTs, which were retrieved and screened from 5 to 10 databases. Each SR included in the analysis consisted of 5 to 23 RCTs, with 433 to 1926 participants. The main interventions of the treatment group are acupuncture (n = 12)^[17,22–32]^ and auricular therapy (n = 4),^[22,24,25,29]^ while the control group mainly receives conventional Western medicine, with the most commonly used medications being Tiapride hydrochloride, Haloperidol, Clonidine, Aripiprazole, and Risperidone. Three studies^[17,24,31]^ used psychological BT as an adjunctive treatment. Ten SRs/MAs^[17,22,23,25–28,30–32]^ used the Cochrane risk of bias tool for assessing methodological quality. Two studies^[24,29]^ used the Jadad scale for assessing methodological quality. The outcome indicators are mainly related to efficacy and safety. Most of the studies included in the SRs/MAs have efficacy outcome indicators such as Response Rate, YGTSS scores, etc, and safety indicators include adverse events incidence.

**Table 1:** Basic information of the studies.

First author (year) country	Search date	No. of searched databases	No. of included RCTs (sample size)	Type of intervention	Comparator	Quality assessment tool	Conclusions that were quoted from the original article	Adverse events
Yu (2016) China^[31]^	March, 2016	10 DBs	5 (433)	AT/PBT	CWM/PBT	Cochrane	“…Acupuncture may have better short-term effect than Western medicine for TS…”	Yes
Lu^[17]^ (2021) China	April, 2020	6 DBs	22 (1668)	AT/PBT	CWM/PBT	Cochrane	“…Acupuncture treatment alone is more effective in the treatment of TD than pharmaceutical treatment…”	Yes
Chung (2016) Korea^[32]^	August, 2016	7 DBs	19 (1483)	AT	CWM	Cochrane	“…This analysis provided limited evidence from studies for the practice of acupuncture in treating TS…”	Yes
Zhu (2020) China^[29]^	October, 2018	5 DBs	19 (1544)	AT/APT	CWM	Jadad	“…Acupuncture and moxibustion is effective in the treatment of Tourette syndrome. Compared with oral Western medicine, it has certain curative effect advantages…”	No
Li (2022) China^[22]^	May, 2021	6 DBs	18 (1380)	AT/APT/Moxa	CWM	Cochrane	“…In the clinical treatment of TD, acupuncture + ear points is the first choice…”	Yes
Zhang (2021) China^[25]^	October, 2020	5 DBs	20 (1596)	AT/APT	CWM	Cochrane	“…Traditional Chinese medicine has a significant therapeutic effect on acupoint selection based on syndrome differentiation…”	Yes
Lin (2022) China^[23]^	January, 2022	7 DBs	14 (905)	AT	CWM	Cochrane	“…General acupuncture may have superior efficacy and safety relative to conventional western medicine for TD…”	Yes
Zhou (2021) China^[28]^	December, 2019	7 DBs	8 (597)	AT	CWM	Cochrane	“…Current evidence supports that the effect of scalp acupuncture treatment on Tourette Syndrome is better than that of Western drubs…”	Yes
Zhao (2020) China^[27]^	December, 2018	7 DBs	10 (703)	AT	CWM	Cochrane	“…The results showed that acupuncture had a certain curative effect on TD…”	No
Ni (2017) China^[24]^	May, 2016	7 DBs	23 (1926)	AT/APT/PBT	CWM/PBT	Jadad	“…Acupuncture can be used as a monotherapy or adjuvant therapy to conventional therapy…”	Yes
Zhao (2016) China^[26]^	February, 2015	9 DBs	8 (555)	AT	CWM	Cochrane	“…Acupuncture can increase Tourette syndrome total effective than medicine…”	Yes
Pu (2023) China^[30]^	November, 2022	8 DBs	17 (1276)	AT	CWM	Cochrane	“…compared with Western medicine commonly used in clinical practice, acupuncture and acupuncture combined with tuina therapy have better effects on improving TD in children…”	Yes

APT = auricular point therapy, AT = acupuncture, CWM = conventional Western medicine, DB = databases, Moxa = moxibustion, PBT = psychobehavioral therapy.

### 3.3. AMSTAR-2 assessment of quality

The results of the AMSTAR-2 evaluation are shown in Table 2. Eleven SRs/MAs are of low quality, and 1 is of very low quality. All authors of the studies have developed research protocols, but only 2 studies have registered protocols (item 2). All SRs explained the reasons for the selection of study types (item 3), with only one study utilizing a comprehensive search strategy, while the rest of the studies had some deficiencies in their search strategies (item 4). However, no SRs provided a complete list of excluded studies and their reasons (item 7). All SRs used appropriate tools to assess the risk of bias (item 9). None of the studies reported the funding sources of the included studies (item 10). All SRs employed appropriate statistical methods to combine the results of the studies (item 11). Only 1 SR did not consider the risk of bias in the included studies (item 13) and did not assess publication bias (item 15), while 4 studies did not report any potential sources of conflict of interest (item 16).

**Table 2:** Results of quality evaluation with AMSTAR-2.

First author (year)	Q1	Q2	Q3	Q4	Q5	Q6	Q7	Q8	Q9	Q10	Q11	Q12	Q13	Q14	Q15	Q16	Ranking of quality
Zhu (2020)^[29]^	Y	PY	Y	PY	N	Y	N	PY	Y	N	Y	N	Y	N	Y	N	Low
Li (2022)^[22]^	Y	PY	Y	PY	N	Y	N	PY	Y	N	Y	N	Y	N	Y	N	Low
Zhang (2021)^[25]^	Y	PY	Y	PY	N	Y	N	PY	Y	N	Y	N	Y	Y	Y	N	Low
Lin (2022)^[23]^	Y	PY	Y	PY	N	Y	N	PY	Y	N	Y	N	Y	Y	Y	N	Low
Ni (2017)^[24]^	Y	PY	Y	PY	N	Y	N	PY	PY	N	Y	N	Y	Y	Y	N	Low
Zhou (2021)^[28]^	Y	PY	Y	PY	N	Y	N	PY	Y	N	Y	N	Y	N	Y	N	Low
Zhao (2020)^[27]^	Y	PY	Y	PY	N	Y	N	PY	Y	N	Y	N	Y	Y	Y	N	Low
Lu (2021)^[17]^	Y	Y	Y	PY	N	Y	N	Y	Y	N	Y	N	Y	Y	Y	Y	Low
Yu (2016)^[31]^	Y	PY	Y	PY	N	Y	N	Y	Y	N	Y	N	N	Y	N	Y	Critically low
Chung (2016)^[32]^	Y	PY	Y	PY	N	Y	N	PY	Y	N	Y	N	Y	Y	Y	Y	Low
Zhao (2016)^[26]^	Y	PY	Y	Y	N	Y	N	PY	Y	N	Y	N	Y	Y	Y	N	Low
Pu (2023)^[30]^	Y	Y	Y	PY	N	Y	N	PY	Y	N	Y	N	Y	N	Y	Y	Low

Important items 2, 4, 7, 9, 11, questions 13 and 15 are of very low quality if 2 or more of these items are omitted, low quality if 1 important item is omitted, moderate quality if 2 or more of the noncritical items other than the above important items are omitted, no missing items or non-important If 1 item is omitted, it is rated as excellent quality.

Domains:

1. Did the research questions and inclusion criteria for the review include the components of PICO?

2. Did the report of the review contain an explicit statement that the review methods were established prior to the conduct of the review and did the report justify any significant deviations from the protocol?

3. Did the review authors explain their selection of the study designs for inclusion in the review?

4. Did the review authors use a comprehensive literature search strategy?

5. Did the review authors perform study selection in duplicate?

6. Did the review authors perform data extraction in duplicate?

7. Did the review authors provide a list of excluded studies and justify the exclusions?

8. Did the review authors describe the included studies in adequate detail?

9. Did the review authors use a satisfactory technique for assessing the risk of bias (RoB) in individual studies that were included in the review?

10. Did the review authors report on the sources of funding for the studies included in the review?

11. If meta-analysis was performed did the review authors use appropriate methods for statistical combination of results?

12. If meta-analysis was performed, did the review authors assess the potential impact of RoB in individual studies on the results of the meta-analysis or other evidence synthesis?

13. Did the review authors account for RoB in individual studies when interpreting/discussing the results of the review?

14. Did the review authors provide a satisfactory explanation for, and discussion of, any heterogeneity observed in the results of the review?

15. If they performed quantitative synthesis did the review authors carry out an adequate investigation of publication bias (small study bias) and discuss its likely impact on the results of the review?

16. Did the review authors report any potential sources of conflict of interest, including any funding they received for conducting the review?

AMSTAR-2 = A Measurement Tool to Assess Systematic Reviews-2, N = no, PY = partially yes, Y = yes.

### 3.4. Quality of evidence evaluation

After examining 64 outcomes, none had high-quality evidence. According to the grading assessment, there were 24 outcomes with moderate-quality evidence, 30 outcomes with low-quality evidence, and 10 outcomes with very low-quality evidence, as shown in Table 3.

**Table 3:** Results of quality of evidence evaluation with GRADE approach.

First author (year)	Outcomes	Number of RCTs (number of participants)	Relative absolute (95% CI)	*P*-value	Quality of evidence
Zhu (2020)^[29]^	YGTSS-overall score	8 (742)	MD −4.55 [−6.74, −2.39]	<.0001	Low
	Overall response rate	19 (1530)	OR 2.49 [1.89,3.27]	<.00001	Low
Lin (2022)^[23]^	YGTSS-overall score	5 (320)	SMD −1.63 [−1.89, −1.36]	<.00001	Moderate
	YGTSS (BA and CN VS CWM)	1 (80)	SMD −0.54 [−0.99, −0.1]	.02	Moderate
	YGTSS (BA vs CWM)	1 (60)	SMD −2.29 [−2.95, −1.63]	<.00001	Moderate
	YGTSS (CN vs CWM)	3 (180)	SMD −2.18 [−2.55, −1.8]	<.00001	Moderate
	Overall response rate	14 (699)	RR 1.29 [1.19,1.39]	<.00001	Low
	Response rate (BA and CN VS CWM)	7 (479)	RR 1.25 [1.14,1.38]	<.00001	Moderate
	Response rate (BA vs CWM)	3 (186)	RR 1.3 [1.1,1.54]	.003	Moderate
	Response rate (CN vs CWM)	4 (240)	RR 1.36 [1.15,1.61]	.0003	Moderate
Zhao (2020)^[27]^	YGTSS-overall score	6 (424)	SMD −0.71 [−1.1, −0.33}	.0003	Low
	Overall response rate	10 (703)	RR 1.15 [1.05,1.25]	.002	Very low
	Response rate (BA vs CWM)	1 (64)	RR 1.11 [0.96,1.28]	.17	Very low
	Response rate (CN vs CWM)	5 (340)	RR 1.2 [1.04,1.38]	.02	Low
	Response rate (BA and CN vs CWM)	4 (299)	RR 1.14 [0.97,1.33]	.11	Very low
	Response rate (AT vs CWM)	7 (519)	RR 1.14 [1.03,1.27]	.01	Low
	Response rate (EA vs CWM)	3 (184)	RR 1.18 [0.95,1.47]	.13	Very low
	Marked response rate	10 (703)	RR 1.30 [1.13,1.50]	.0002	Low
	Marked response rate (BA and CN vs CWM)	4 (299)	RR 1.30 [1.01,1.67]	.04	Low
	Marked response rate (CN vs CWM)	5 (340)	RR 1.27 [1.01,1.6]	.04	Low
	Marked response rate (BA vs CWM)	1 (64)	RR 1.5 [1.13,1.99]	.005	Low
Zhou (2021)^[28]^	YGTSS-overall score (CN vs CWM)	6 (564)	SMD −1.31 [−1.81, −0.81]	<.00001	Low
	Overall response rate (CN vs CWM)	9 (558)	OR 2.89 [1.85,4.51]	<.00001	Low
	Adverse events incidence (CN vs CWM)	2 (100)	RR 0.63 [0.41,0.98]	.04	Moderate
Ni (2017)^[24]^	Overall response rate	22 (1543)	OR 2.94 [2.24,3.85]	<.00001	Low
	YGTSS-overall score	5 (420)	SMD −1.29 [−1.87, −0.7]	<.0001	Low
	YGTSS-motor tic	5 (325)	SMD −0.34 [−0.78,0.09]	.12	Very low
	YGTSS-vocal tic	5 (420)	SMD −1.24 [−1.81, −0.68]	<.000 1	Low
	YGTSS-impairment	5 (420)	SMD −1.08 [−1.29, −0.87]	<.000 01	Low
Zhang (2021)^[25]^	Overall response rate	20 (1596)	OR 3.12 [1.95,4.98]	<.00001	Very low
	Response rate (AT vs CWM)	12 (943)	OR 3.81 [2.17,6.68]	<.00001	Very low
	Response rate (APT vs CWM)	5 (653)	OR 1.74 [0.95,3.19]	.07	Very low
	Adverse events incidence	9 (745)	OR 0.06 [0.03,0.13]	<.00001	Low
Yu (2016)^[31]^	YGTSS-overall score	2 (180)	MD −4.6 [−5.8, −3.4]	<.00001	Moderate
	Response rate	5 (429)	RR 1.19 [1.08,1.31]	.0006	Moderate
Lu (2021)^[17]^	YGTSS-overall score	15 (1171)	MD −2.79 [−4.75, −0.82]	.005	Low
	YGTSS (AT vs CWM)	12 (930)	MD −2.36 [−4.74, −0.02]	.05	Very low
	YGTSS (EA vs CWM)	3 (241)	MD −3.83 [−5.32, −2.34]	<.00001	Low
	Overall response rate	22 (1668)	RR 1.14 [1.09,1.20]	<.00001	Low
	Response rate (AT vs CWM)	19 (1427)	RR 1.15 [1.09,1.21]	<.00001	Low
	Response rate (EA vs CWM)	3 (241)	RR 1.11 [1.01,1.23]	.04	Moderate
	Adverse events incidence	7 (640)	RR 0.26 [0.17,0.41]	<.00001	Moderate
	Recurrence rate	6 (405)	RR 0.28 [0.17,0.46]	<.00001	Moderate
Chung (2016)^[32]^	Response rate	18 (1297)	RR 1.17 [1.1,1.25]	<.00001	Low
	YGTSS-overall score	3 (260)	MD 3.46 [−0.67,7.62]	.1	Low
Zhao (2016)^[26]^	YGTSS-overall score	3 (180)	MD −5.62 [−7.24, −4]	<.00001	Low
	YGTSS-motor tic	3 (180)	MD −0.21 [−0.83,0.42]	.52	Very low
	YGTSS-vocal tic	3 (180)	MD −2.03 [−2.4, −1.66]	<.00001	Moderate
	YGTSS-impairment	3 (180)	MD −4.1 [−5.1, −3.09]	<.00001	Low
	Overall response rate	8 (555)	RR 1.2 [1.1,1.32]	<.0001	Low
	Response rate (AT vs CWM)	4 (274)	RR 1.23 [1.06,1.43]	.007	Moderate
	Response rate (EA vs CWM)	3 (222)	RR 1.18 [1.04,1.35]	.01	Moderate
Pu (2023)^[30]^	Overall response rate	39 (3038)	OR 1.72 [1.29,2.30]	<.05	Moderate
	YGTSS-motor tic	24 (1767)	MD 3.96 [1.75,6.17]	<.05	Moderate
	YGTSS-vocal tic	21 (1626)	MD 3.40 [2.58,4.22]	<.05	Moderate
	YGTSS-impairment	21 (1649)	MD 5.95 [4.23,7.68]	<.05	Moderate
	TCM syndrome-nod and shrug	9 (712)	MD 1.70 [1.15, 2.26]	<.05	Moderate
	TCM syndrome-vocalization in the larynx	10 (804)	MD 1.50 [1.06, 1.95]	<.05	Moderate
	TCM syndrome-upset	10 (666)	MD 1.17 [0.62, 1.73]	<.05	Moderate
	TCM syndrome-main disease	15 (1174)	MD 3.17 [2.41, 3.93]	<.05	Moderate
Li (2022)^[22]^	Overall response rate	18 (1380)	OR 1.82 [1.07,3.11]	<.05	Low
	Adverse events incidence (AT vs CWM)	6 (380)	OR 0.16 [0.05,0.48]	<.05	Low
	Adverse events incidence (AT and Moxa vs CWM)	2 (128)	OR 0.05 [0.01,0.42]	<.05	Low
	Adverse events incidence (AT and APT vs CWM)	3 (262)	OR 0.22 [0.02,0.78]	<.05	Low

CI = confidence interval, APT = auricular point therapy, AT = acupuncture, BA = body acupuncture, CN = cephalic needle, CWM = conventional Western medicine, EA = electroacupuncture, GRADE = grading of recommendations assessment, development, and evaluation, Moxa = moxibustion, OR = odds ratio.

### 3.5. Effectiveness of acupuncture for TD

All SRs summarized the evidence of acupuncture as a treatment for TD, as presented in Table 3. Evidence from 12 SRs, encompassing 64 outcomes collectively, suggested that acupuncture is more effective than conventional Western medication for treating TD. Except for Li Jianrong,^[22]^ all SRs reported a Response Rate, while only Zhang Yingying^[25]^ did not present YGTSS scores. Regarding Response Rate and YGTSS scores, the meta-analysis indicated a statistically significant difference between the AT group and the control group. One study^[30]^ compared traditional Chinese symptom scores and found that AT treatment achieved significantly greater efficacy compared to the control group. Another study^[27]^ reported remission rates and found that the AT group exhibited superior efficacy compared to the control group. A study^[17]^ reported recurrence rates, indicating a lower rate of recurrence in TD patients after AT treatment. Across all SRs, the most commonly used acupuncture methods were conventional needling, followed by scalp acupuncture, electroacupuncture, and ear acupressure. Three SRs^[24,31,32]^ documented acupoint selection for acupuncture treatment of TD, with the optimal acupoints being GV20 (Baihui), GB20 (Fengchi), followed by GV24 (Shenting), LR3 (Taichong), and EX-HN1 (Sishencong).

### 3.6. Adverse events

Among all the included studies, 4 trials^[17,22,25,28]^ (comprising 19 outcomes) provided evidence supporting the lower incidence of adverse reactions in the acupuncture group compared to the group receiving Western medicine, 10 studies^[17,22–26,28,30–32]^ addressed adverse reactions associated with acupuncture treatment for TD, while only 2 studies^[27,29]^ did not mention this aspect. Adverse reactions mainly include drowsiness, fatigue, dry mouth, nausea, constipation, subcutaneous bleeding, etc. Only 1 study^[24]^ mentioned dizziness. Although there were no serious adverse events directly related to acupuncture, due to the small sample size and some missing data in the included review, we cannot fully confirm the safety of acupuncture.

## 4. Discussion

This review evaluated all the available evidence regarding the use of AT for TD. The AMSTAR-2 tool was utilized to assess the methods and quality of the included studies, and the GRADE tool was employed to evaluate the quality of the evidence. This study incorporated 12 SRs, comprising a total of 106 RCTs involving 7973 participants.

Based on the evidence included in this study, the use of AT for TD has demonstrated clinical benefits. However, all the SRs evaluated by AMSTAR-2 exhibited at least 1 critical flaw and several noncritical flaws, resulting in the categorization of all SRs as being of low or very low quality. Out of the 12 SRs assessed, 11 were rated as low quality, while 1 was rated as very low quality. Regarding the key items (2, 4, 7, 9, 11, 13, 15), the absence of a comprehensive list documenting the excluded studies and their corresponding reasons for exclusion (specifically highlighted in item 7) contributed to the overall downgrading of the quality of the SRs. This downgrading was primarily driven by the deficiencies observed in the non-key items (1, 3, 5, 6, 8, 10, 12, 14, 16), such as the failure to conduct study selection in duplicate (item 5), failure to report on the funding sources for the studies included in the review (item 10), and lack of assessments addressing the potential impact of bias in individual studies on the results of the meta-analysis, as conducted by the authors of the SRs (item 12).

Although most RCTs support the use of AT for TD, the quality of these RCTs is generally poor, leading to the low quality of SRs. AMSTAR-2 is merely an assessment tool and cannot substitute for comprehensive judgment of the results of SRs. Additionally, the reliability and validity of AMSTAR-2 also require further validation.

We conducted a GRADE evaluation of 64 outcome measures from 12 studies. Among these, 10 outcomes were rated as very low-quality evidence, 30 as low-quality evidence, and 24 as moderate-quality evidence, with none rated as high-quality evidence, indicating an overall low level of evidence quality. The primary reason for evidence downgrading in the GRADE assessment was study limitations (risk of bias). The majority of RCTs failed to conceal allocation and implement blinding, leading to this outcome. It is noteworthy that, according to the GRADE rating, the study by Tong et al^[30]^ demonstrated that AT could improve symptoms such as nod and shrug, vocalization in the larynx, and upset in patients with TD, with the evidence quality rated as moderate. This indicates that AT has the potential to improve TCM Syndrome in TD patients and is worthy of clinical recommendation. However, in aspects such as response rate, incidence of adverse events, and YGTSS score, the GRADE rating primarily indicates moderate to low-quality evidence. This suggests that further research is needed to verify the clinical efficacy and safety of AT in the treatment of TD.

The most frequently utilized acupoints are GV20 (Baihui) on the Governor Vessel meridian and GB20 (Fengchi) on the Gallbladder Meridian of Foot-Shaoyang, followed by GV24 (Shenting), LR3 (Taichong) on the Liver Meridian of Foot-Jueyin, and the extra point EX-HN1 (Sishencong). In clinical practice, children with TS are predominantly diagnosed with the Liver Hyperactivity with Wind Stirring pattern.^[33]^, which likely informs the primary rationale for acupoint selection. GV20 is often paired with GB20 to calm the mind, regulate spirit, and harmonize Qi flow. Needling techniques typically employ filiform needles (diameter 0.25–0.30 mm): GV20, GV24 and EX-HN1 receive subcutaneous insertion (depth 10–15 mm), GB20 is obliquely inserted toward the nasal tip (depth 10–20 mm), and LR3 undergoes perpendicular insertion (depth 5–10 mm). Needles are retained for 20 to 30 minutes, with treatments administered 3 to 5 times weekly over 8 to 12 weeks.^[34–37]^ Research indicates^[38]^ that acupuncture at GV20 and GV24 alleviates tic symptoms in murine models by inhibiting dopamine receptor expression.

During our research, we found that certain SRs/MAs focusing on the combined use of AT and CWM for the treatment of TD demonstrated superior therapeutic effects compared to the use of CWM alone. Due to the scarcity of relevant SRs/MAs studies, we exclusively included SRs/MAs that compared the use of AT alone versus CWM for the treatment of TD in order to minimize potential biases and enhance the credibility of the evidence. Consequently, it is anticipated that in the future, there will be SRs/MAs assessing the efficacy of combined therapy with AT and CWM for treating TD, thereby advancing the establishment of clinical evidence.

The clinical management of TD has become increasingly diversified, with AT offering distinctive advantages in alleviating tic symptoms among affected children. Literature reports demonstrate that the combination of AT and TCM yields superior treatment outcomes for TD. Additionally, AT can effectively serve as an adjunctive therapeutic approach to enhance the efficacy of CWM. Consequently, AT stands as a preferred choice among clinicians, affected children, and their families in the clinical management of TD.

## 5. Strength and limitations

This study represents the first overview of reviews on the use of AT in the treatment of TD. All studies included in this overview were RCTs. To mitigate the risk of bias, we developed and registered a study protocol in advance. We established stringent quality assessment criteria and employed AMSTAR-2 to evaluate the methodological quality of each SR/MA. Additionally, we utilized GRADE to assess the quality of evidence for AT outcomes in TD treatment, aiming to enhance the validity and reliability of the study results. However, it is important to note that this overview has several limitations. Firstly, the overall poor quality of the included SRs/MAs undoubtedly diminishes the credibility of this overview. Furthermore, we did not conduct a comprehensive search and detailed analysis of the original data from the RCTs, potentially leading to the omission of biases within the included SRs/MAs. Therefore, caution should be exercised when interpreting the conclusions derived from this overview.

## 6. Conclusions

Although there is evidence that AT has advantages in treating TD, the methodological quality of most studies is low, which limits our ability to draw definitive conclusions. Large-sample, multicenter clinical studies are needed to be performed so as to establish solid evidence and make further recommendations.

Supplemental digital content “ PRISMA_2020_checklist, PRISMA_flow diagram, Excluded document and Search strategy” are available for this article (http://links.lww.com/MD/Q903).

## Author contributions

**Conceptualization:** Xiaoqian Li, Lei Xu, Lisheng Wan, Guozheng Qin.

**Data curation:** Xiaoqian Li, Lei Xu.

**Formal analysis:** Xiaoqian Li, Lei Xu.

**Funding acquisition:** Xiaoqian Li, Lei Xu.

**Investigation:** Xiaoqian Li, Lei Xu.

**Methodology:** Xiaoqian Li, Lei Xu.

**Project administration:** Xiaoqian Li, Lei Xu, Huiling Liang.

**Resources:** Xiaoqian Li, Lei Xu, Huiling Liang.

**Software:** Xiaoqian Li, Lei Xu, Huiling Liang.

**Supervision:** Xiaoqian Li, Lei Xu, Lisheng Wan, Guozheng Qin.

**Validation:** Xiaoqian Li, Lei Xu.

**Visualization:** Xiaoqian Li, Lei Xu, Jinlan Peng.

**Writing – original draft:** Xiaoqian Li, Lei Xu.

**Writing – review & editing:** Xiaoqian Li, Lei Xu, Lisheng Wan, Guozheng Qin.

## Supplementary Material
